# Orf virus early genes ORFV113 and ORFV114 are individually dispensable for replication in vitro but required for full virulence in vivo

**DOI:** 10.1186/s13567-026-01877-w

**Published:** 2026-11-10

**Authors:** Weijie Zhou, Jingjin Hu, Xiaoyue Wang, Naixiu Wang, Zhentao Cheng, Anchun Cheng, Feng Pang

**Affiliations:** Institute of Veterinary Immunology and Green Drugs, Department of Veterinary Medicine, College of Animal Science, and State Key Laboratory of Green Pesticide, Guizhou University, Guiyang, 550025 China

**Keywords:** Orf virus, ORFV113 gene, ORFV114 gene, deletion mutants, replication kinetics, pathogenicity

## Abstract

Orf is a highly contagious zoonotic disease caused by Orf virus (ORFV), resulting in significant economic losses to the livestock industry. *ORFV113* and *ORFV114*, located in the terminal variable region of the ORFV genome, are putative virulence-associated genes. To elucidate their biological functions, we characterized the transcription kinetics of both genes and generated single-gene deletion mutants by homologous recombination, using transfer plasmids carrying flanking homology arms and an enhanced green fluorescent protein (EGFP) reporter cassette. Lamb testis (LT) cells transfected with the recombinant plasmids were infected with the parental ORFV-JS strain; recombinant viruses were enriched by limiting dilution, purified by consecutive rounds of plaque isolation, and verified by polymerase chain reaction (PCR). Both genes are highly conserved among ORFV isolates (92–100% nucleotide identity) and are transcribed with early kinetics. The genetically stable deletion mutants, designated ORFV-Δ113 and ORFV-Δ114, were successfully generated. Deletion of either gene did not significantly alter viral growth kinetics or cytopathic effects (CPE) in LT cells. In a rabbit skin scarification model (*n* = 4 per group), however, both mutants induced markedly milder macroscopic lesions with lower cumulative clinical scores (*P* < 0.05), and viral loads in scab tissue were reduced 17-fold (ORFV-Δ113) and 10-fold (ORFV-Δ114) relative to the wild-type strain (*P* < 0.01). Thus, ORFV113 and ORFV114 are dispensable for viral replication in vitro but are required for full virulence in vivo. The resulting deletion mutants provide valuable tools for dissecting virus–host interactions and are promising candidates for the rational design of live-attenuated ORFV vaccines.

## Introduction

Contagious ecthyma (commonly known as Orf) is a highly contagious, zoonotic, and epitheliotropic skin disease that primarily affects sheep and goats worldwide [1, 2]. Characterized by ulcerative, pustular, and scabby lesions predominantly at mucocutaneous junctions, the disease severely impairs animal feeding. This leads to significant weight loss and stunted growth, inflicting substantial economic losses on the sheep and goat industry [3–5].

The etiological agent, Orf virus (ORFV), belongs to the genus *Parapoxvirus* within the family *Poxviridae* [6]. As an epitheliotropic virus, ORFV completes its entire replication cycle within the cytoplasm of host cells [7]. Its genome consists of approximately 130–140 kb of linear double-stranded DNA, encompassing 132 open reading frames (ORFs) [8, 9]. Structurally, the genome comprises a highly conserved central core (ORFs 009–111), which predominantly encodes genes involved in viral DNA replication and maturation. This core region is flanked by highly variable terminal regions (ORFs 001–008 and 112–134), which mainly encode genes associated with viral virulence, inflammatory responses, and immunomodulation [10, 11]. ORFV possesses a remarkable reinfection capacity, which is fundamentally attributed to the multifaceted immune evasion mechanisms encoded in its terminal variable regions [12]. These mechanisms are predominantly mediated by a repertoire of viral immunomodulatory proteins (IMPs) that target distinct nodes of the host immune defense network [13, 14]. Well-characterized IMPs include a chemokine-binding protein (CBP; ORFV112) [15], the anti-apoptotic protein ORFV125 [16], a viral interleukin (IL)-10 homolog (ORFV127) with high sequence homology to host IL-10 [17], an inhibitor of granulocyte–monocyte colony-stimulating factor and IL-2 (ORFV117) [18], and a vascular endothelial growth factor homolog (ORFV132) that drives vascular proliferation [19, 20]. Accordingly, ORFV113 and ORFV114, located within the right terminal variable region, are hypothesized to participate in viral virulence and host immunomodulation.

We previously generated and characterized a mouse polyclonal antibody against the truncated recombinant ORFV113 protein [21], and established an indirect enzyme-linked immunosorbent assay (ELISA) based on the highly conserved recombinant ORFV114 protein [22]. Despite these preliminary investigations, the precise biological functions of ORFV113 and ORFV114 remain unclear. To investigate their roles, we generated their respective single-gene deletion mutants (ORFV-Δ113 and ORFV-Δ114) via homologous recombination. We characterized their in vitro replication kinetics through 50% tissue culture infectious dose (TCID_50_) titration, cytopathic effect (CPE) profiling, and multistep growth curve analyses. Furthermore, in vivo virulence profiles were evaluated utilizing a New Zealand White rabbit dorsal scarification model, coupled with the quantification of viral genomic loads within infected scab tissues. Collectively, these investigations elucidate the respective contributions of ORFV113 and ORFV114 to viral replication and pathogenesis, providing an experimental framework for unraveling the complex immunomodulatory networks of ORFV.

## Materials and methods

### Cells, viruses, and plasmids

HeLa cells were purchased from Haixing Biosciences (Suzhou, China). Primary lamb testis (LT) cells were prepared and maintained in our laboratory. Both cell types were propagated in high-glucose Dulbecco’s modified Eagle medium (DMEM; Procell, Wuhan, China) supplemented with 10% fetal bovine serum (FBS; Cell-Box) and 1% penicillin–streptomycin (Solarbio, Beijing, China). Cells were maintained in a humidified incubator at 37 °C with 5% CO_2_. *Escherichia coli* DH5α competent cells were purchased from Tsingke Biotechnology (Beijing, China). New Zealand White rabbits were sourced from Guiyang Pengcheng Agricultural Technology Co., Ltd. (Guiyang, China). The parental wild-type ORFV strain (ORFV-JS) [23, 24], the pUC57 cloning vector, and the pEGFP-N1 plasmid were maintained in our laboratory.

### Sequence alignment and conservation analysis

The ORFV113 (GenBank: OQ030228.1) and ORFV114 (GenBank: OR100514.1) sequences of the ORFV-JS strain were utilized as references. Homologous sequences from 20 representative ORFV strains were retrieved using the National Center for Biotechnology Information (NCBI) Basic Local Alignment Search Tool (BLAST). Sequence alignments and homology analyses between ORFV-JS and the selected reference strains were performed using the MegAlign module within DNASTAR software.

### Transcriptional kinetics analysis

The HeLa cell line was used to investigate the transcriptional kinetics of ORFV113 and ORFV114, because it is a well-validated, highly permissive model for studying the temporal transcriptional cascade of poxviruses. Briefly, HeLa cells were inoculated with the ORFV-JS (multiplicity of infection [MOI] = 1) and assigned to three conditions: cytarabine‑treated infected cells (AraC^+^; 40 μg/mL) to suppress viral DNA replication, untreated infected cells (AraC^−^), and mock‑infected cells. Total RNA was harvested at 2, 4, 6, 12, and 24 hours post‑infection (hpi) using the RNAeasy Animal RNA Isolation Kit (Beyotime, Shanghai, China). Following extraction, total RNA was reverse-transcribed into cDNA using the HiScript III 1st Strand cDNA Synthesis Kit (Vazyme, Nanjing, China). Semiquantitative real-time polymerase chain reaction (RT-PCR) amplification was conducted using 2 × PrimeSTAR Mix (Takara, Dalian, China) with target-specific primers. The PCR cycling conditions comprised an initial denaturation at 95 °C for 3 min; 30 cycles of denaturation at 95 °C for 30 s, annealing at the primer-specific temperature for 30 s, and extension at 72 °C for 1 min; and a final extension at 72 °C for 5 min. The established early gene ORFV024 and the late gene ORFV059 were amplified simultaneously as temporal-expression controls [25]. Additionally, the cellular housekeeping gene GAPDH was amplified as an internal reference control to ensure equal template input across all samples. All PCR primers (Table 1) were synthesized by Tsingke Biotechnology Co., Ltd. (Beijing, China).

**Table 1:** **RT-PCR primers used for assessing the transcription kinetics of ORFV113 and ORFV114**

Primer name	Sequence 5′–3′	Product (bp)	Annealing temperature (°C)
ORFV113RT-F	AATATGCCTAACCGGAGCGG	472	57
ORFV113RT-R	GTACGGACCTTGTTGGTCGT		
ORFV114RT-F	ATGCCGCTGTTCCGGAAGCTC	289	58
ORFV114RT-R	CCGATCGCGTACACACGAGC		
ORFV024RT-F	GCGGACACAGCCACAACCACAGTC	420	65
ORFV024RT-R	CTAGCACGCGCTTTCGGTACCGCC		
ORFV059RT-F	ATGGATCCACCCGAAATCAC	1011	60
ORFV059RT-R	TCACACGATGGCCGTGACCAGC		
GAPDH RT-F	ATTCCACCCATGGCAAATTCC	250	58
GAPDH RT-R	ATGACGAACATGGGGGCATC		

### Construction of recombinant transfer plasmids

To construct recombinant transfer vectors for the deletion of ORFV113 and ORFV114, a seamless cloning strategy was employed to insert three fragments—the left homology arm, an enhanced green fluorescent protein (EGFP) expression cassette, and the right homology arm—into a pUC57 vector linearized with *Hin*dIII and *Bam*HI. To ensure robust cytoplasmic expression of EGFP in infected cells, a synthetic poxvirus early/late (pE/L) promoter was incorporated into the forward primers (EGFP/113-F and EGFP/114-F) and fused immediately upstream of the EGFP coding sequence. Overlapping sequences were introduced at the ends of all primers to facilitate the multifragment assembly. All primer sequences are listed in Table 2.

**Table 2:** **Primers used to construct homologous recombination plasmids**

Primer name	Sequence 5′–3′	Product (bp)	Annealing temperature (°C)	Amplicon
113L-F	GACCATGATTACGCCAAGCTTATGAAGGCGGTGTTGTTGCT	864	60	ORFV113 left homology arm
113L-R	TTTAATTGCCAGGGTTGAGGTTAA			
113R-F	CAAGTAAATGCCGCTGTTCCGGAAG	1041	60	ORFV113 right homology arm
113R-R	ATGCATCTAGATATCGGATCCTCAGGGTCCGTCAAGGGC			
114L-F	GACCATGATTACGCCAAGCTTATGGAAGTGCTGGTGATCATCTC	627	63	ORFV114 left homology arm
114L-R	TTCAATTTTTCTACTCGTCGTAGAAAAATTCTCCC			
114R-F	CTGTACAAGTAAATGGCGTGTTTTCTCGAATTG	450	64	ORFV114 right homology arm
114R-R	ATGCATCTAGATATCGGATCCTCATTCCGAGGAGGAGAAGGT			
EGFP/113-F	CCTCAACCCTGGCAATTAAAAAAATTGAAATTTTATTTTTTTTTTTTG	760	60	EGFP cassette (ORFV-Δ113)
EGFP/113-R	GGAACAGCGGCATTTACTTGTACAGCTCGTCCATGCC			
EGFP/114-F	CGACGAGTAGAAAAATTGAAATTTTATTTTTTTTTTTTG	760	60	EGFP cassette (ORFV-Δ114)
EGFP/114-R	CACGCCATTTACTTGTACAGCTCGTCCATGCC			

Underlined sequences denote *Hind*III and *Bam*HI restriction sites, respectively

The homology arms and the EGFP fragment were amplified using 2 × Phanta UniFi Master Mix (Vazyme). The linearized pUC57 vector and the three corresponding fragments were then assembled using the ClonExpress Ultra One Step Cloning Kit (Vazyme) as directed by the manufacturer. The assembly products were transformed into *E. coli* DH5α competent cells, and positive clones were selected on ampicillin-containing agar plates. After screening by restriction digestion and Sanger sequencing, correct clones were expanded, and the plasmids were purified via the EndoFree Plasmid Midi Kit (Tiangen, Beijing, China) for use in cell transfection.

### Generation of ORFV-Δ113 and ORFV-Δ114 deletion mutants

LT cells seeded in six-well plates were transfected with 2.5 μg of the sequence-verified recombinant plasmids using Lipofectamine 3000 (Thermo Fisher Scientific, Waltham, MA, USA), according to the manufacturer’s instructions. At 6 h post-transfection, the cells were infected with the wild-type ORFV-JS strain at an MOI of 1 to initiate homologous recombination. At 48 h post-infection, cells exhibiting strong green fluorescence were harvested via manual scraping and subjected to three freeze–thaw cycles at −80 °C to release intracellular virions. The resulting cell lysate was centrifuged at 3000 rpm for 10 min to remove cellular debris, yielding a crude virus-containing supernatant.

### Purification of recombinant deletion mutants

Recombinant deletion mutants were isolated through a two-stage purification process. First, limiting dilution was performed by serially diluting the crude viral supernatant (10^–1^−10^−6^) in serum-free DMEM and infecting LT cell monolayers in 96-well plates. Following a 2-h adsorption and a 72-h incubation in maintenance medium (DMEM with 2% FBS), wells displaying a single, distinct EGFP-positive focus at dilutions of 10^−3^ or higher were harvested and subjected to repeated freeze–thaw cycles. This limiting dilution process, consisting of five consecutive steps per round, was repeated for three independent rounds to achieve high enrichment of the recombinant viruses.

To ensure genetic purity, the enriched viral supernatants were then further purified by plaque isolation: the supernatants were serially diluted 10^−1^–10^−5^ in serum-free DMEM and inoculated into LT cell monolayers in 12-well plates. After a 2-h viral adsorption period, the inoculum was removed, and the cells were overlaid with a 1:1 mixture of 2% low-melting-point agarose (Sangon Biotech, Shanghai, China) and DMEM supplemented with 4% FBS. Following a 72-h incubation at 37 °C, well-isolated EGFP-positive viral plaques were visually identified and carefully picked using a micropipette. One complete purification round was defined as five consecutive plaque isolation steps, and this purification procedure was repeated for 3–4 independent rounds until no parental virus was detectable.

### PCR identification and genetic stability assays

Viral genomic DNA was extracted from the fully purified ORFV-Δ113 and ORFV-Δ114 deletion mutants. PCR verification was performed utilizing specific primer pairs targeting the ORFV113 and ORFV114 genes and the viral B2L reference gene, with the corresponding primer sequences listed in Table 3; the EGFP expression cassette was amplified using the primer pairs specified in Table 2. Genomic DNA derived from the deletion mutants and the parental ORFV-JS served as templates. To assess viral genetic stability, the purified mutant strains were serially passaged for ten passages in LT cells. Viral genomic DNA was isolated at passages 1, 5, and 10, followed by PCR amplification of the EGFP gene to confirm the stable integration and retention of the exogenous reporter sequence.

**Table 3:** **Primers used for PCR identification and genetic-stability analysis of the ORFV113 and ORFV114 deletion mutants**

Primer name	Sequence 5′–3′	Product (bp)	Annealing temperature (°C)
ORFV113-F	CCAAGCTGGCTAGTTAAGCTTATGGAAGTGCTGGTGATCATCTC	627	60
ORFV113-R	TGCTGGATATCTGCAGAATTCCTCGTCGTAGAAAAATTCTCCCTC		
ORFV114-F	CCAAGCTGGCTAGTTAAGCTTATGCCGCTGTTCCGGAAG	1041	60
ORFV114-R	TGCTGGATATCTGCAGAATTCGGGTCCGTCAAGGGCGTC		
B2L-F	CTAGCGTTTAAACTTAAGCTTATGTGGCCGTTCTCCTCCA	1137	60
B2L-R	TGCTGGATATCTGCAGAATTCATTTATTGGCTTGCAGAACTCCG		

### Viral titration (TCID_50_) and cytopathic effects evaluation

Viral titers were determined utilizing the 50% tissue culture infectious dose (TCID_50_) assay. LT cell monolayers in 96-well plates were infected with 10-fold serial dilutions 10^−1^–10^−10^ of either the wild-type or mutant viruses, utilizing eight replicate wells per dilution. During a 5–7-day incubation period, wells exhibiting characteristic CPE and green fluorescence were monitored and recorded daily using an inverted fluorescence microscope. Final TCID_50_ values were calculated using the Reed–Muench method and expressed as log_10_ TCID_50_ per 0.1 mL. To compare cytopathic characteristics, LT cells in 12-well plates were infected with the ORFV-JS, ORFV-Δ113, or ORFV-Δ114 strains at an MOI of 1. The progression of CPE, predominantly characterized by cell rounding and detachment, was microscopically monitored and documented at 48 and 72 hpi.

### Viral growth curves

LT cell monolayers in 12-well plates were infected with the ORFV-JS, ORFV-Δ113, or ORFV-Δ114 strains at an MOI of 0.1. At designated time points (6, 12, 24, 36, 48, and 72 hpi), the viral suspensions (comprising both cells and medium) were harvested. The collected samples were subjected to three freeze–thaw cycles at −80 °C and subsequently centrifuged to clarify the supernatants. The infectious viral titers within the cleared supernatants were quantified at each time point via TCID_50_ assays on LT cells, as previously described. Viral multistep growth kinetics were plotted using GraphPad Prism 8 software (GraphPad Software, San Diego, CA, USA), with time post-infection represented on the *x*-axis and log_10_TCID_50_/0.1 mL values on the *y*-axis. All viral titer values represent the means ± standard deviations (SD) derived from three independent biological replicates.

### In vivo pathogenicity evaluation of deletion mutants

One-month-old New Zealand White rabbits (1.0–1.2 kg, male; *n* = 16) were confirmed to be negative for ORFV antigen and ORFV-specific antibodies by indirect ELISA and were then randomly allocated into four groups (*n* = 4 per group). Following scarification of the shaved dorsal skin, rabbits were inoculated with 10^5^ TCID_50_ of the respective viral strains (ORFV-JS, ORFV-Δ113, or ORFV-Δ114) via topical application to the abraded skin surface, while the control group received an equivalent volume of sterile DMEM via the same administration route. Clinical scoring was performed every 3 days over a 21-day observation period to evaluate macroscopic lesions, including erythema and crusting, according to a modified scoring matrix adapted from Zhu et al. [26] (Table 4). At 14 days post-infection (dpi), 30 mg of scab tissue was aseptically collected from each infected group, and genomic DNA was extracted using the Tissue Genomic DNA Extraction Kit (Tiangen, Beijing, China). Viral DNA loads were quantified by amplifying the conserved B2L gene using the 2 × Hieff Unicon qPCR TaqMan Probe Master Mix (Yeasen, Shanghai, China) on a CFX96 Real-Time PCR Detection System (Bio-Rad, Hercules, CA, USA). The specific experimental procedures were performed with reference to the detection methods previously established by our research group [27]. The primers and TaqMan probe used for B2L gene detection were designed with the following nucleotide sequences (5′–3′): the forward primer B2L-F is GCTCTACTCCACCAACAA, the reverse primer B2L-R is GCGAGTCCGAGAAGAATA, and the fluorescent probe B2L-P is HEX-ATGAACCGCTACAACACCTTCTACT-BHQ1, with the HEX reporter dye labeled at the 5′ terminus and the BHQ1 quencher labeled at the 3′ terminus. For this assay, the standard‑curve regression equation is Ct = −3.4762log_10_ (copy number) + 43.203 (R^2^ = 0.9966, E = 94%).

**Table 4:** **Clinical scoring matrix for skin lesions in rabbits inoculated with ORFV-JS, ORFV-Δ113, and ORFV-Δ114**

Scoring dimensions	Score 0	Score 1	Score 2	Score 3
Lesion severity	No visible lesions	Local redness and swelling	Typical papules or vesicles	Pustules, ulcers, or evident scabs
Lesion area	Absent	< 5 mm	5–10 mm	>10 mm or confluent lesions
Healing status	Normal healing	Slow progression	Progressive worsening	Secondary infection or necrosis

### Statistical analysis

All quantitative analyses were conducted via IBM SPSS Statistics 27.0. Differences in clinical lesion scores among groups were evaluated using a one-way analysis of variance (ANOVA) followed by Dunnett’s T3 post hoc test. An independent-samples *t*-test was employed to assess differences in viral loads between each individual deletion mutant and the parental wild-type strain. Statistical significance was defined as *P* < 0.05, and high statistical significance was designated as* P* < 0.01.

## Results

### ORFV113 and ORFV114 are highly conserved early genes during ORFV infection

**Figure 1:**
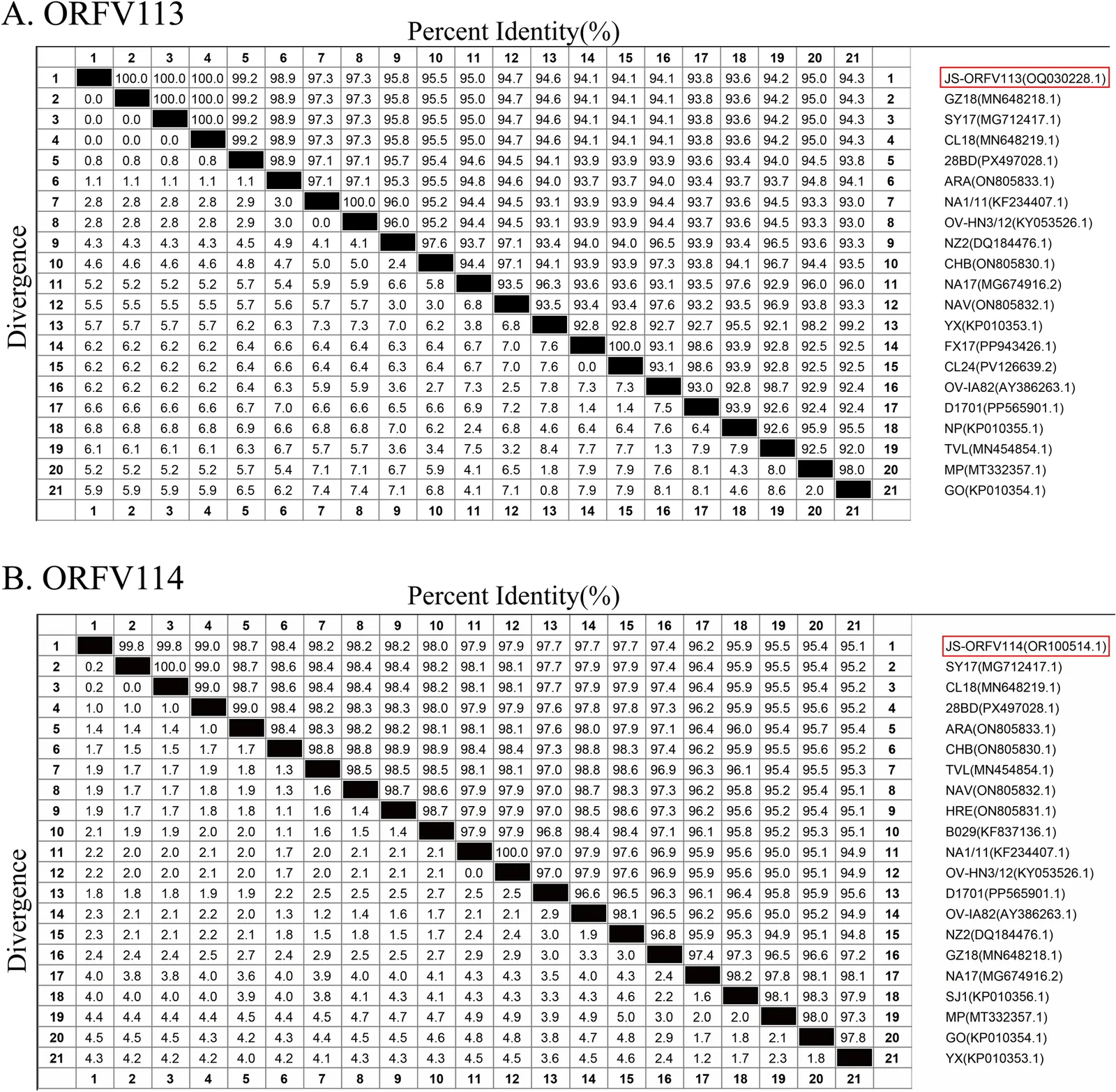
**Nucleotide sequence homology analysis of ORFV113 (A) and ORFV114 (B).** Nucleotide identity matrices illustrate the pairwise nucleotide similarity of the ORFV113 and ORFV114 genes, respectively, generated from sequence alignments between the ORFV-JS strain and 20 representative ORFV reference strains.

Sequence alignment revealed that the ORFV113 and ORFV114 genes of the ORFV-JS strain are highly conserved, sharing 92–100% nucleotide identity with the corresponding sequences from 20 representative ORFV strains (Figure 1). Notably, homology reaches 100% for ORFV113 and 99.8% for ORFV114 when compared specifically with the local Chinese isolates GZ18, CL18, and SY17. These data establish ORFV113 and ORFV114 as highly conserved elements within the ORFV genome.

**Figure 2:**
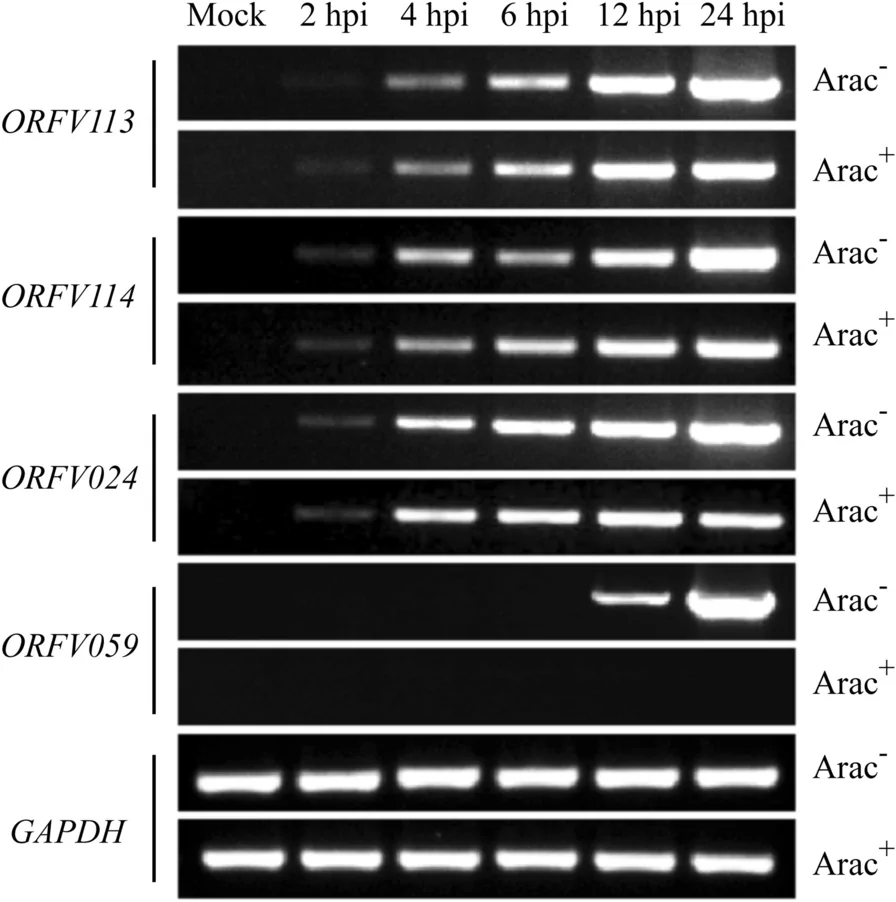
**Transcriptional kinetics of ORFV113 and ORFV114 genes during ORFV infection.** Semiquantitative RT-PCR analysis of ORFV113 and ORFV114 transcription at 2, 4, 6, 12, and 24 h post-infection. The viral early gene ORFV024 and late gene ORFV059 were included as temporal expression controls, with GAPDH as the internal control. Data are representative of three independent biological replicates. AraC^+^: AraC treated; AraC^−^: untreated; mock: uninfected control.

RT-PCR analysis demonstrated that ORFV113 and ORFV114 transcripts were detectable as early as 2 hpi in both AraC-treated (AraC^+^) and untreated (AraC^−^) groups, with expression levels increasing progressively up to 24 hpi (Figure 2). Crucially, the transcription of both genes was not blocked by AraC, an inhibitor of viral DNA replication. This transcriptional kinetic profile closely mirrored that of the early reference gene, ORFV024. Conversely, transcripts of the late reference gene, ORFV059, were only detected in the untreated group starting at 12 hpi. Meanwhile, the cellular housekeeping gene GAPDH exhibited stable and comparable band intensities across all time points and both AraC-treated and untreated groups, confirming equal template loading and consistent reverse transcription efficiency for all samples. Together, these findings classify ORFV113 and ORFV114 as highly conserved early genes during ORFV infection.

### Generation of EGFP-reporter ORFV-Δ113 and ORFV-Δ114 deletion mutants

**Figure 3:**
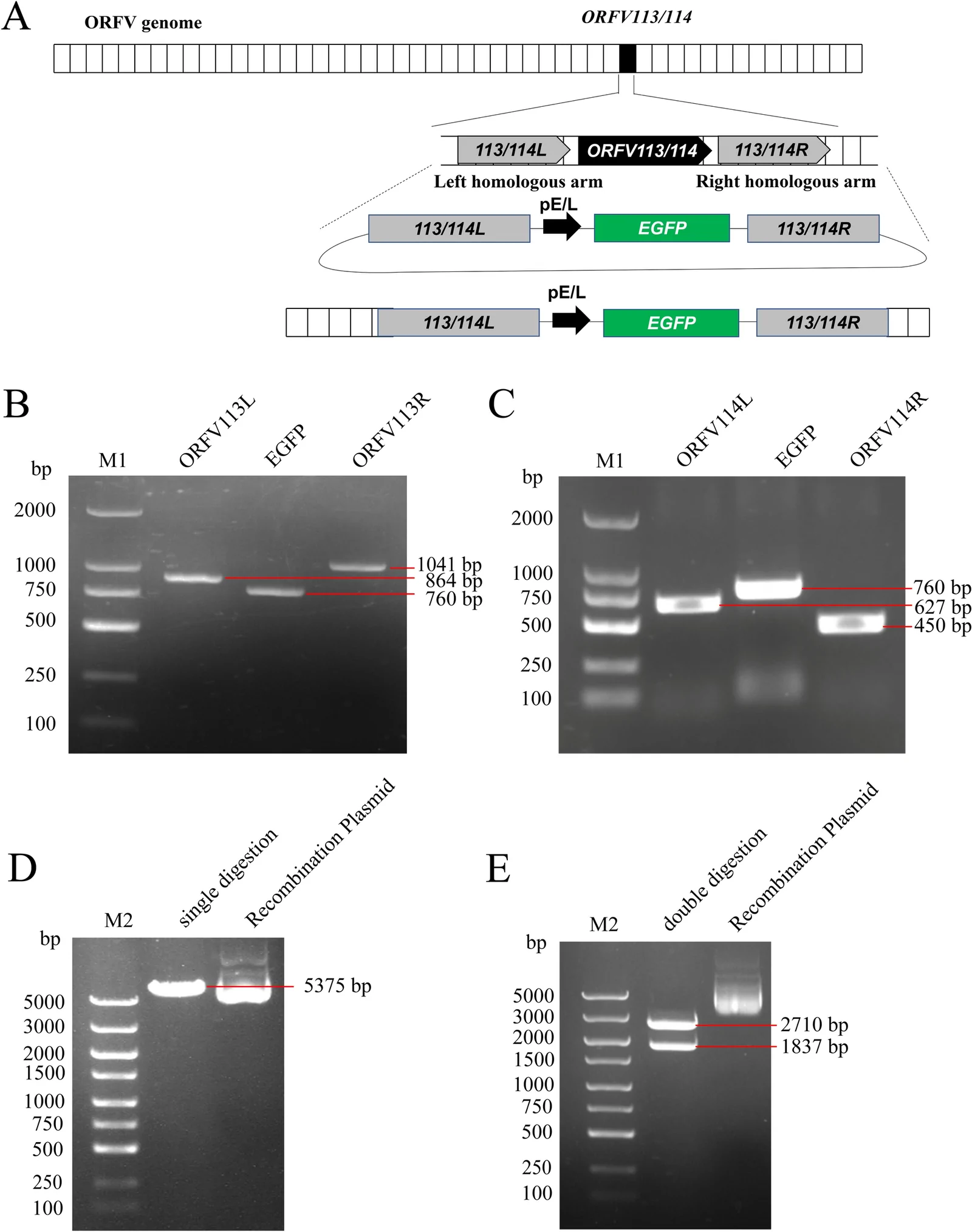
**Construction and verification of the recombinant transfer plasmids.** **A** Schematic representation of the homologous recombination strategy for target gene deletion and EGFP reporter cassette insertion. **B**, **C** PCR amplification of the left homologous arms (ORFV113L, ORFV114L), the EGFP reporter cassette, and the right homologous arms (ORFV113R, ORFV114R). **D**, **E** Restriction enzyme digestion analysis (single digestion for pUC57-EGFP-Δ113 and double digestion for pUC57-EGFP-Δ114). M1: DL2000 DNA ladder; M2: DL5000 DNA ladder.

Following the strategy for generating the ORFV-Δ113 and ORFV-Δ114 deletion mutants shown in Figure 3A, target fragments were amplified using the ORFV-JS genome and the pEGFP-N1 plasmid as templates. Agarose gel electrophoresis confirmed the successful amplification of the left and right homologous arms for ORFV113 (864 bp and 1041 bp) and ORFV114 (627 bp and 450 bp), along with the EGFP expression cassette (approximately 760 bp) (Figure 3B, C). The identity of the pUC57-EGFP-Δ113 construct was confirmed via *Hin*dIII single digestion, which yielded the expected 5375 bp linearized band (Figure 3D). Similarly, the pUC57-EGFP-Δ114 plasmid was verified via *Hin*dIII and *Bam*HI double digestion, producing the anticipated 2710 bp and 1837 bp fragments (Figure 3E). Finally, Sanger sequencing verified the seamless assembly of both recombinant plasmids, confirming the absence of spurious mutations in the homologous arms or the reporter cassette.

**Figure 4:**
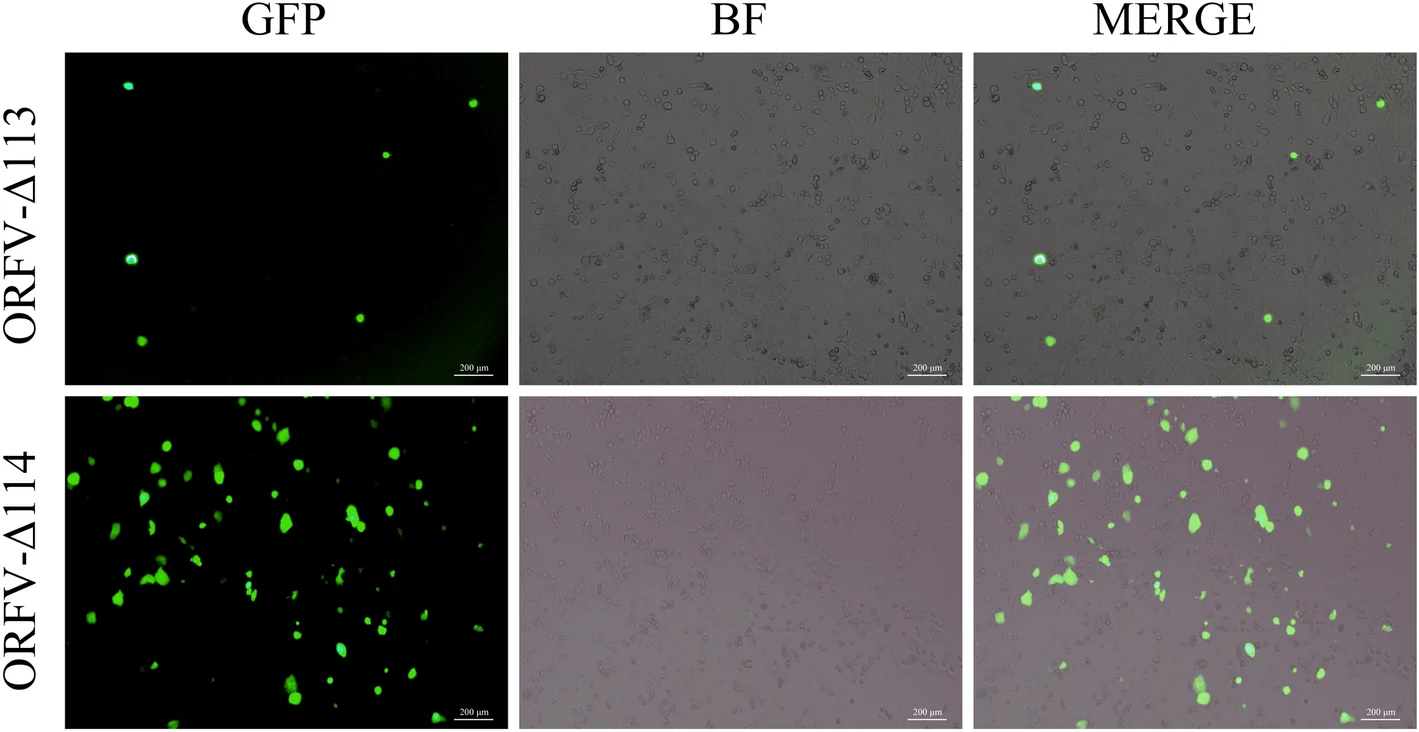
**Generation of the ORFV-Δ113 and ORFV-Δ114 deletion mutants.** Upper panel: ORFV-Δ113 deletion mutants; lower panel: ORFV-Δ114 deletion mutants. GFP: EGFP fluorescence channel indicating reporter gene expression and recombinant virus replication; BF: bright-field channel showing virus-induced cellular cytopathic effects; MERGE: overlay of GFP and bright-field channels.

As shown in Figure 4, LT cells were transfected with the recombinant transfer plasmids and subsequently infected with the wild-type ORFV-JS strain. At 48 hpi, discrete green fluorescent foci were detected by fluorescence microscopy. The spatial overlap of EGFP fluorescence with CPE foci was consistent with successful homologous recombination. The resulting crude recombinant viral lysates, designated as ORFV-Δ113 and ORFV-Δ114, were harvested and cryopreserved at −80 °C for further purification.

### Purification of ORFV-Δ113 and ORFV-Δ114 deletion mutants

**Figure 5:**
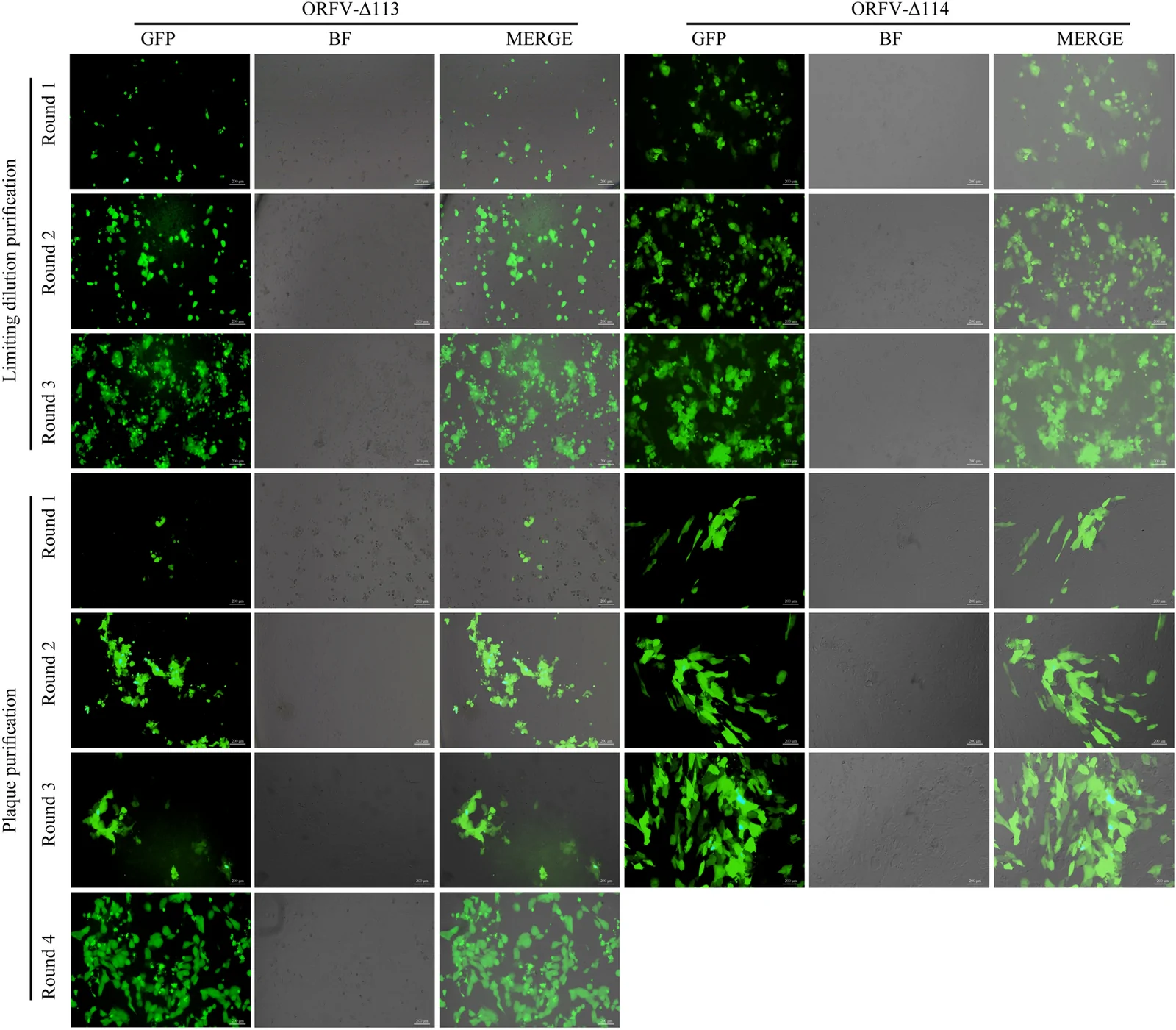
**Purification of the ORFV-Δ113 and ORFV-Δ114 deletion mutants.** Upper panel: progressive enrichment of recombinant viruses via three consecutive rounds of limiting dilution purification. Lower panel: further purification to genetic homogeneity via three to four consecutive rounds of plaque isolation under low-melting-point agarose overlay. For each purification stage, the ORFV-Δ113 mutant is shown on the left and the ORFV-Δ114 mutant on the right, with images captured in EGFP fluorescence (GFP), bright-field (BF), and merged (MERGE) channels.

Following three consecutive rounds of limiting dilution, the proportion of EGFP-positive cells within the infected monolayers progressively increased (Figure 5, upper panel). Notably, the spatial distribution of the fluorescent signals largely overlapped with localized areas of cytopathic effects (CPE). This indicated the continuous enrichment of recombinant virions and a significant reduction in the non-fluorescent, wild-type parental virus background. To achieve genetic purity, the enriched viral populations were subsequently subjected to plaque purification under a low-melting-point agarose overlay. After three to four rounds of stringent plaque isolation, the resulting viral progeny exhibited uniform EGFP expression that completely overlapped with the CPE foci (Figure 5, lower panel). This phenotypic consistency confirmed that the ORFV-Δ113 and ORFV-Δ114 deletion mutants had been successfully purified to homogeneity, with no detectable parental virus.

### Verification and genetic stability of ORFV-Δ113 and ORFV-Δ114 deletion mutants

**Figure 6:**
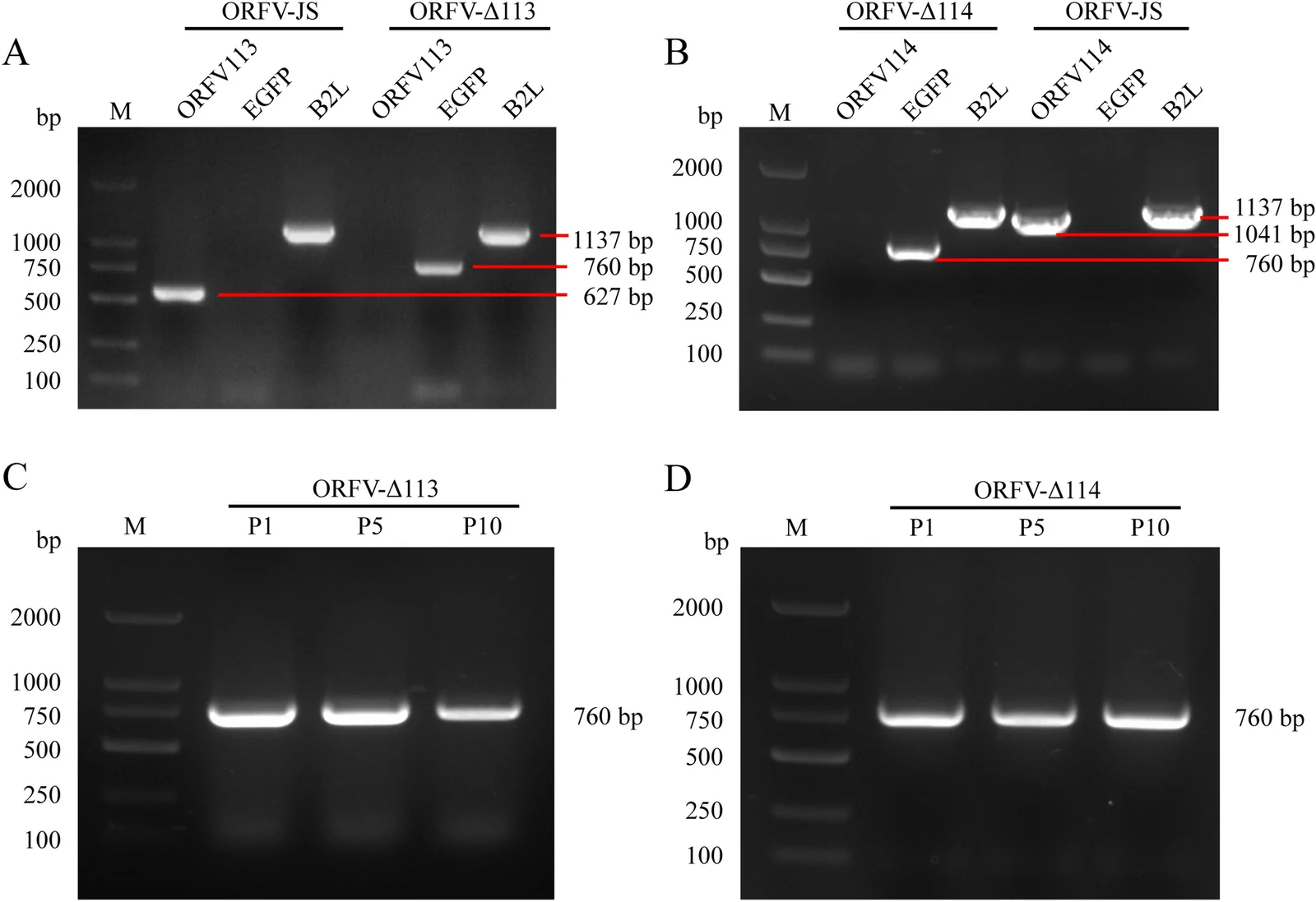
**PCR validation and genetic stability of ORFV-Δ113 and ORFV-Δ114 deletion mutants.** **A**, **B** PCR validation of the ORFV-Δ113 (**A**) and ORFV-Δ114 (**B**) deletion mutants. Genomic DNA from the purified deletion mutants and parental ORFV-JS was analyzed for the presence of the endogenous target genes, the inserted EGFP cassette, and the B2L reference gene. **C**, **D** Genetic stability assessment. The stable genomic retention of the EGFP cassette in the ORFV-Δ113 (**C**) and ORFV-Δ114 (**D**) deletion mutants was verified by PCR amplification across serial passages (P1, P5, and P10). M: DL2000 DNA ladder.

PCR-based molecular characterization verified the targeted gene deletions in the mutant strains (Figure 6A, B). As expected, the wild-type ORFV-JS yielded amplicons for B2L (1137 bp) and the target genes (ORFV113, 627 bp; ORFV114, 1041 bp) but not EGFP. Conversely, ORFV-Δ113 and ORFV-Δ114 each yielded an approximately 760-bp EGFP reporter cassette and a 1137-bp B2L amplicon. The endogenous ORFV113 amplicon was absent from ORFV-Δ113, whereas the endogenous ORFV114 amplicon was absent from ORFV-Δ114. These results confirmed the successful gene replacement in the ORFV-Δ113 and ORFV-Δ114 deletion mutants. Furthermore, in vitro genetic stability assays demonstrated that the 760-bp EGFP amplicon was consistently detected across passages 1, 5, and 10 in LT cells, confirming the stable integration and genomic retention of the exogenous expression cassette over continuous viral replication (Figure 6C, D).

### ORFV113 and ORFV114 are dispensable for ORFV replication in vitro

**Figure 7:**
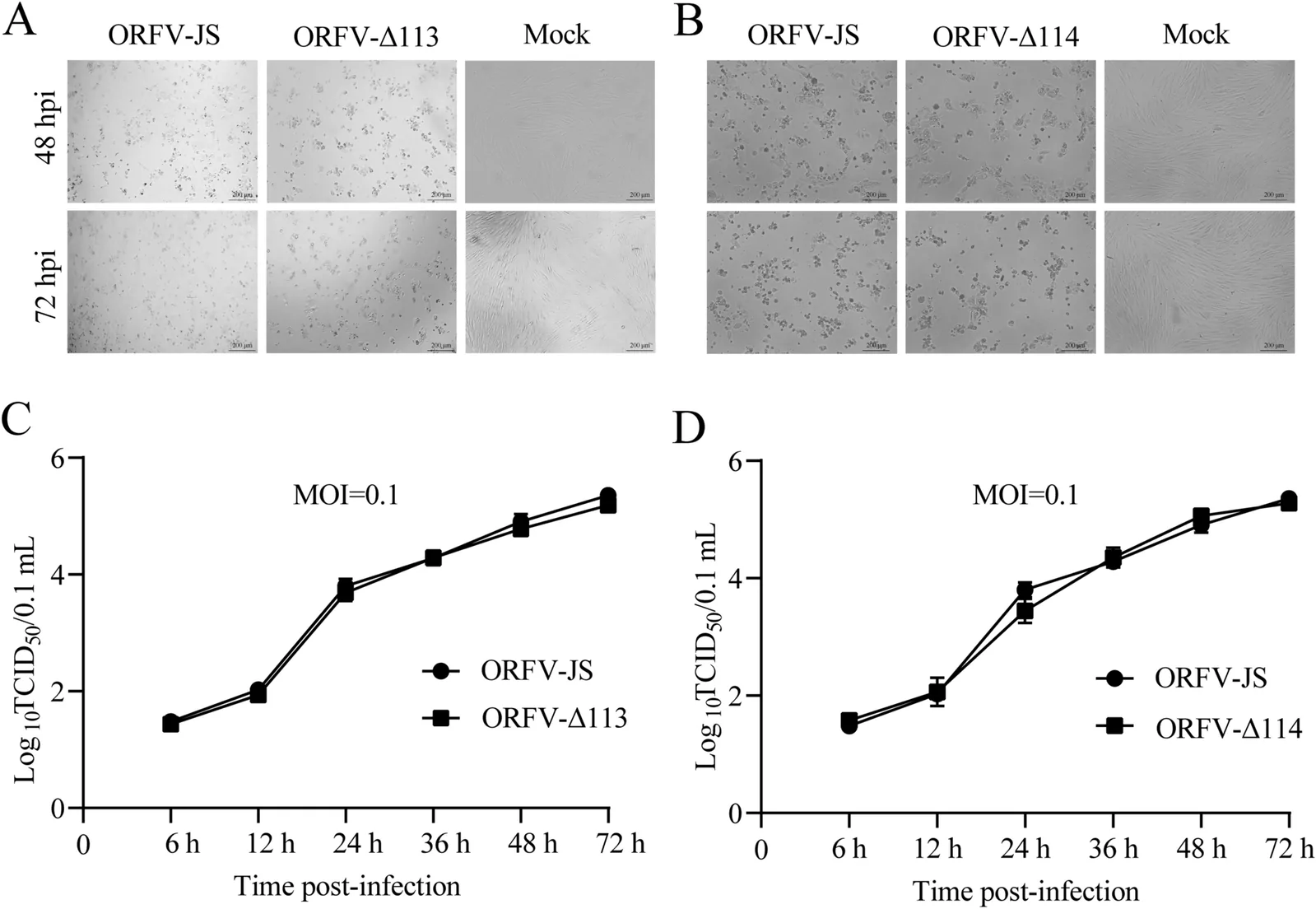
**Cytopathogenicity and in vitro replication kinetics of the deletion mutants.** **A**, **B** Microscopic observation of cytopathic effects (CPE) induced by the ORFV-Δ113 (**A**) and ORFV-Δ114 (**B**) deletion mutants compared with the parental ORFV-JS strain at the indicated time points post-infection. **C**, **D** Multistep in vitro growth curves detailing the viral replication trajectories of the ORFV-Δ113 (**C**) and ORFV-Δ114 (**D**) deletion mutants versus the parental ORFV-JS strain in LT cells over a 72-h period.

We first examined whether the deletion mutants retained the ability to induce cytopathic effects in LT cells. Microscopic evaluation at 48 and 72 hpi revealed that both ORFV-Δ113 and ORFV-Δ114 elicited characteristic CPE, including cell shrinkage, rounding, and detachment—morphologically indistinguishable from those caused by the parental ORFV-JS strain (Figure 7A, B), whereas mock-infected cells maintained normal spindle-shaped morphology.

We next quantified the replication efficiency of the deletion mutants. Viral titers determined by the Reed–Muench method showed that both deletion mutants exhibited infectious titers comparable to those of the parental ORFV-JS strain, with no significant differences observed. Specifically, the wild-type ORFV-JS reached 10^5.359^ TCID_50_/0.1 mL, whereas the ORFV-Δ113 and ORFV-Δ114 deletion mutants achieved titers of 10^5.189^ and 10^5.118^ TCID_50_/0.1 mL, respectively. To further assess viral replication capacity, we performed multistep growth curve analyses in LT cells inoculated with equal infectious doses of each virus (Figure 7C, D). The replication kinetics of ORFV-Δ113 and ORFV-Δ114 in LT cells closely paralleled those of the parental strain from 6 to 72 h post-infection, with all viruses reaching a replication plateau at approximately 72 hpi. Collectively, these data demonstrate that neither ORFV113 nor ORFV114 is required for efficient viral replication or the induction of typical cytopathic effects in vitro.

### Deletion of ORFV113 or ORFV114 significantly attenuates ORFV pathogenicity in vivo

**Figure 8:**
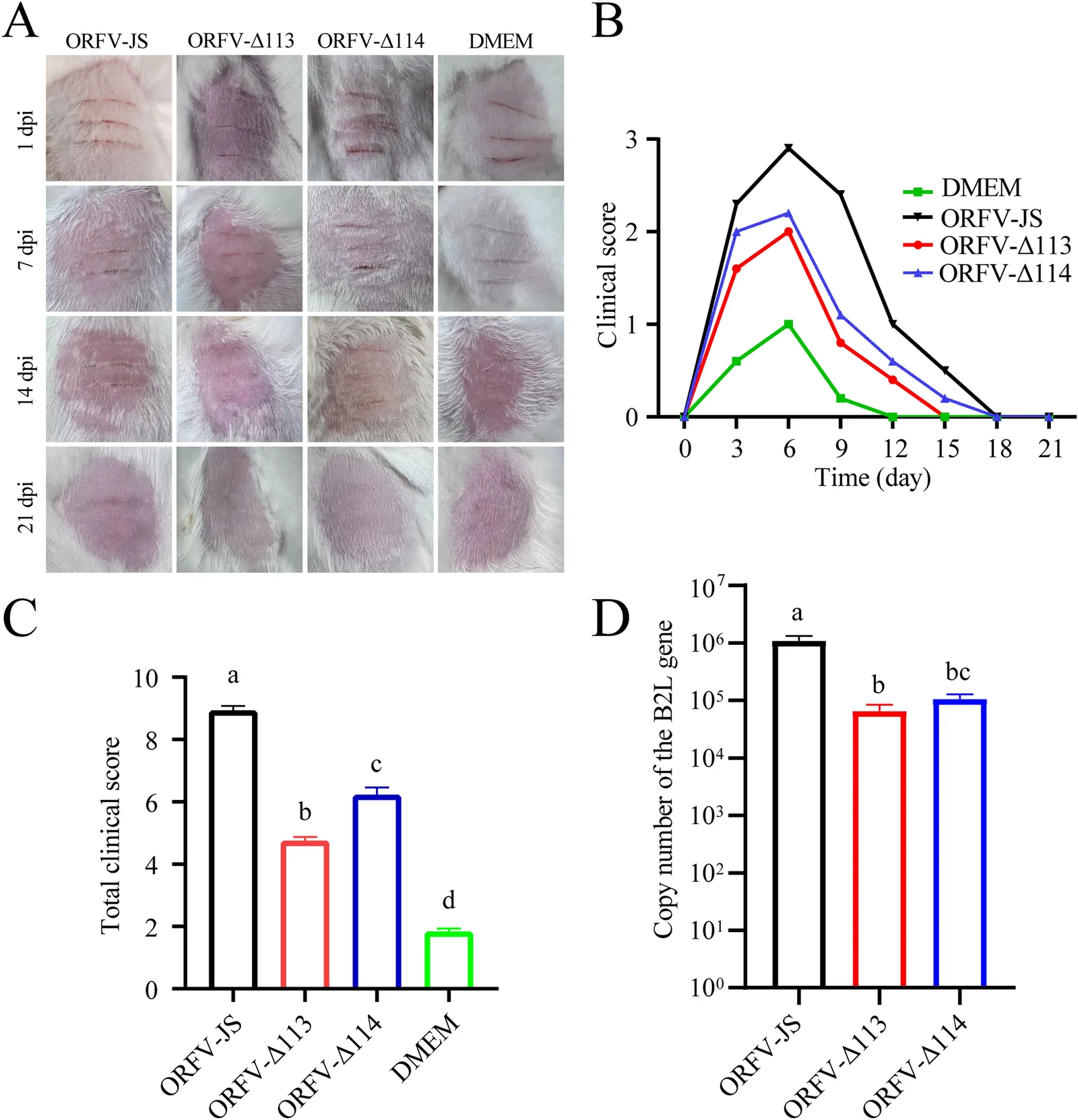
**In vivo pathogenicity evaluation of ORFV deletion mutants in a rabbit skin scarification model.** **A** Macroscopic progression of skin lesions at 1, 7, 14, and 21 days post-inoculation (dpi) in groups challenged with ORFV-JS, ORFV-Δ113, ORFV-Δ114, and DMEM (mock control). **B** Time course of clinical scores recorded at 3-day intervals over the 21-day observation period. **C** Cumulative clinical scores of each group across the full observation period. **D** Viral genomic loads in scab tissues at 14 dpi quantified by TaqMan qPCR targeting the conserved B2L gene. Different lowercase letters indicate statistically significant differences (*P* < 0.05), while identical letters denote no statistical significance.

In the New Zealand White rabbit skin scarification model, the parental ORFV-JS strain induced typical, localized macroscopic lesions over the 21-day observation period, characterized by severe erythema, edema, and robust crust formation (Figure 8A). Conversely, rabbits inoculated with either the ORFV-Δ113 or ORFV-Δ114 mutant exhibited a delayed and attenuated pathological progression, resulting in significantly reduced cumulative clinical scores (*P* < 0.05) (Figure 8A–C). Furthermore, TaqMan-based qPCR quantification of viral genomic loads in scab tissues harvested at 14 dpi revealed a highly significant reduction in viral replication in vivo (Figure 8D). Relative to the wild-type ORFV-JS, the viral DNA loads in the ORFV-Δ113 and ORFV-Δ114 groups were reduced by approximately 17-fold and 10-fold, respectively (*P* < 0.01). Collectively, these findings demonstrate that the ablation of either ORFV113 or ORFV114 severely attenuates ORFV virulence, with the ORFV-Δ113 mutant displaying a more pronounced degree of attenuation.

## Discussion

The evolutionary success of ORFV is largely attributed to its arsenal of immunomodulatory proteins, which are predominantly encoded within the highly variable terminal regions of its genome. In this study, we demonstrate that ORFV113 and ORFV114 are highly conserved early viral genes that are dispensable for viral replication in vitro but required for full virulence in vivo.

Our in vitro findings for ORFV113 are fully consistent with those of Ning et al. in the IA82 background [28], in which gene deletion likewise left replication kinetics unaltered. Critically, our in vivo data extend this observation by demonstrating that ORFV113 contributes substantially to macroscopic lesion formation and viral tissue burden. These phenotypes align well with a previous report showing that infection of sheep with OV-IA82Δ113 resulted in strikingly attenuated disease and reduced viral loads, further confirming ORFV113 as a critical virulence determinant in vivo [29]. Notably, the present study represents the first construction and characterization of an ORFV114 deletion mutant, revealing a phenotypic profile strikingly similar to that of ORFV-Δ113. Although both mutants are significantly attenuated, the more pronounced reduction in viral load for ORFV-Δ113 (17-fold) compared with ORFV-Δ114 (10-fold) is noteworthy. This quantitative difference implies that ORFV113 might exert a more dominant immunomodulatory effect in vivo, or that these two viral proteins counteract host antiviral networks with varying degrees of potency. This parallel behavior raises intriguing questions about potential functional redundancy, complementary roles in antagonizing distinct immune pathways, or synergistic cooperation in immune evasion. Distinguishing among these mechanisms warrants systematic investigation.

The New Zealand White rabbit skin scarification model serves as a useful and practical surrogate for evaluating ORFV virulence [30, 31]. Utilizing this model, our in vivo evaluation established that the ablation of either gene reduces the severity of macroscopic lesions (manifesting as reduced erythema and delayed crust formation) and markedly diminishes viral genomic loads within infected tissues. Nevertheless, an inherent limitation must be recognized. Because the rabbit is a proxy rather than the natural host, and considering the strict host tropism typical of poxviruses, it remains necessary to validate whether the ORFV-Δ113 and ORFV-Δ114 deletion mutants exhibit identical attenuation profiles and decreased viral shedding in natural ovine and caprine hosts through future controlled trials. Building upon these attenuation phenotypes, such planned in vivo studies will systematically evaluate the immunogenicity, challenge protection, and overall safety of these mutants, thereby determining their true potential as live-attenuated vaccine candidates.

Furthermore, the precise host signaling networks modulated by ORFV113 and ORFV114 to drive viral virulence require further elucidation. To address this, our future investigations will employ an integrated transcriptomic and proteomic approach to systematically decode the altered virus–host interactome mediated by these gene deletions. Through Gene Ontology (GO) and Kyoto Encyclopedia of Genes and Genomes (KEGG) enrichment analyses, we aim to map differentially expressed genes to functional pathways central to viral pathogenesis and antiviral innate immunity. Specifically, we will focus on pathways such as cGAS-STING, Toll-like receptor signaling, NF-κB, and JAK-STAT signaling, with the ultimate goal of pinpointing the critical host regulatory nodes targeted by ORFV113 and ORFV114.

## Conclusions

In this study, we characterized ORFV113 and ORFV114 as highly conserved viral early genes and generated their respective single-gene deletion mutants (ORFV-Δ113 and ORFV-Δ114). Deletion of either gene does not affect viral replication in vitro but significantly reduces pathogenicity in a rabbit scarification model. These findings demonstrate that ORFV113 and ORFV114 are important virulence-associated genes contributing to ORFV pathogenicity in vivo. These genetically defined deletion mutants provide valuable tools for dissecting viral immunomodulatory mechanisms and represent promising candidates for the rational design of live-attenuated ORFV vaccines.

## Data Availability

The datasets generated during and/or analyzed during the current study are available from the corresponding author on reasonable request.
